# Understanding plant resilience by putting photosynthesis and photorespiration in the metabolic context

**DOI:** 10.1007/s00425-026-04961-9

**Published:** 2026-03-05

**Authors:** Berkley J. Walker, Wheaton L. Schroeder

**Affiliations:** 1 Department of Energy Plant Research Laboratory, Michigan State University, East Lansing, MI 48824 USA; 2 Department of Plant Biology, Michigan State University, East Lansing, MI 48824 USA; 3 Gene and Linda Voiland School of Chemical Engineering and Bioengineering, Washington State University, Pullman, WA 99164 USA

**Keywords:** Serine metabolism, Genome-scale modeling, Stress physiology, Energy dissipation, Nitrogen assimilation, One-carbon flux

## Abstract

**Main conclusion:**

Photorespiration is a dynamic metabolic process that contributes to energy balance, stress resilience, and nutrient flux, warranting its integration into genome-scale models to enhance plant productivity and climate adaptation.

**Abstract:**

Photorespiration, sometimes referred to as a wasteful byproduct of rubisco’s oxygenation activity, is increasingly recognized as a vital and multifaceted component of plant metabolism. This perspective explores three underappreciated roles of photorespiration: as an alternative energy sink, a marker of stress resilience, and a metabolic hub. Photorespiration consumes significant ATP and reducing equivalents, potentially serving as a photoprotective mechanism under environmental stress. However, its role in energy dissipation remains debated, particularly in relation to non-photochemical quenching. Stress conditions such as drought and heat elevate photorespiratory flux due to Rubisco kinetics and stomatal responses, yet the link between photorespiration and resilience is complex and species-dependent. Metabolites like serine and glycine, key intermediates in photorespiration, correlate with stress responses and may exit the canonical pathway, contributing to one-carbon metabolism and amino acid biosynthesis. Calculations suggest that serine export from photorespiration could explain nitrate assimilation rates, yet protein synthesis alone cannot account for this flux, indicating unknown metabolic sinks. Genome-scale metabolic models (GSMMs) and resource allocation models (RAMs) offer promising tools to integrate photorespiration into broader metabolic frameworks. These models can simulate open-loop versus closed-loop photorespiration, assess energy dissipation capacity, and track amino acid fate. Future research should focus on refining GSMMs to include accurate photorespiratory pathways and leveraging them to understand photorespiration’s role in plant resilience and nutrition, especially under realistic field conditions. This integrated approach is essential for reimagining photorespiration not as a metabolic burden, but as a central player in plant adaptation and productivity.

## Introduction

Photorespiration is a critical component of plant photosynthesis yet is often seen as a “wasteful” catalytic accident; however, the reality is more nuanced. While photorespiration is critical to plant survival, it is also responsible for massive amounts of leaf energy use and carbon release (Walker et al. 2016; Busch 2020; Sharkey 1988). Photorespiration arose as the solution to the challenge of the substrate specificity of the first enzyme of C3 carbon fixation (ribulose-1,5-bisphosphate carboxylase/oxygenase, rubisco). Rubisco’s photosynthetic role is to carboxylate (add carbon dioxide to) the C3 cycle intermediate ribulose 1,5-bisphosphate to form 3-phosphoglycerate (3PG), which can then enter the C3 cycle to produce sugars. Rubisco does not have perfect specificity for carbon dioxide; however, it also oxygenates (adds molecular oxygen to) ribulose 1,5-bisphosphate, producing the inhibitory molecule 2-phosphoglycolate (2PG). Photorespiration recycles 2PG back into the C3 cycle intermediate 3PG (Bauwe 2023). This recycling detoxifies 2PG, while maintaining most of the previously reduced carbon bonds, but at the cost of energy (ATP and reducing equivalents) and released carbon dioxide (Maurino and Peterhansel 2010).

**Fig. 1:**
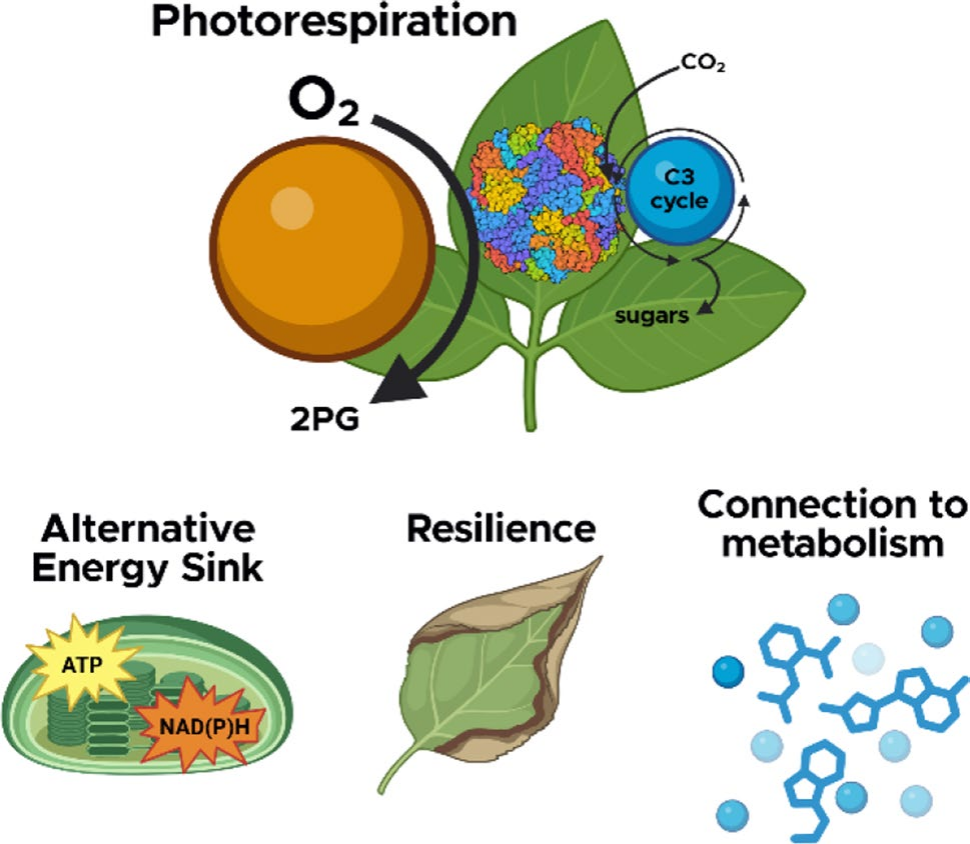
This perspective explores the role of photorespiration in broader plant physiology. Photorespiration starts with the detoxification of 2-phosphoglycolate (2PG) and relates to ATP and NAD(P)H use, plant resilience, and as a hub for metabolism. Image from Biorender

In this perspective we highlight three areas where we see special value for future research in photorespiration. Our purpose is not to review in depth the mechanisms, cover all possible knowledge gaps, or evaluate engineering efforts related to photorespiration, but instead focus on how photorespiration interacts with other aspects of plant physiology and metabolism. These three areas are (1) *the role of photorespiration as an alternative energy sink*, (2) *photorespiration as a marker and key trait for stress resilience*, and (3) *photorespiration in the metabolic context* (Fig. 1)**.** Our hope is that these directions will broaden interest in the study of photorespiration to facilitate an integrated understanding of this fundamental component of photosynthesis.

### The role of photorespiration as an alternative energy sink

Under field conditions, photorespiration is estimated to consume up to 30% of leaf reducing equivalents (NADPH, NADH, and Ferredoxin) and 40% of the ATP produced from photosynthesis (Walker et al. 2016; Sharkey 1988). This large energy use logically connects to a central challenge of the light reactions: excess harvested energy needs to be dissipated to prevent damage to photosynthetic proteins and lipids (photoprotection) (Bassi, et al. 2021). Discussions of photorespiration as a photoprotective energy sink have taken place since the pathway was resolved (Kozaki and Takeba 1996; Wu et al. 1991), and have primarily focused on its demand for reducing equivalents (electron sink).

Evidence for photorespiration serving a photoprotective role through energy dissipation is mixed. On the one hand, there is evidence that photorespiration acts as a photoprotective electron sink under high light, limiting CO_2_, low/high temperatures, and possibly under drought (Heber et al. 1996; Heber and Krause 1980; Manuel et al. 1999; Guan and Gu 2009; Hendrickson et al. 2003; Osei-Bonsu et al. 2021). On the other hand, it has also been argued that this energy consumption is only a small fraction of the total energy dissipated through non-photochemical energy quenching processes, both regulated and unregulated (Bauwe 2023; Osmond and Grace 1995; Pinnola and Bassi 2018). Recent work demonstrates that when photorespiration is minimized (e.g. low oxygen conditions) there was no evidence for photodamage in *Nicotiana tabacum*, even under high light, but instead a possible bottleneck in ATP turnover leading to an increased activation of rapidly reversible non-photochemical quenching (q_e_) (Smith et al. 2024). This form of non-photochemical quenching is not associated with photodamage, presenting an alternative connection between photorespiration and photoprotection. Photorespiration as an “ATP sink” is an especially interesting prospect, since it consumes ATP at a higher ratio than the C3 cycle (Walker et al. 2020; Kramer and Evans 2011).

These studies indicate that future work examining the role of photorespiration in energy dissipation should focus on which energy species (ATP or reducing equivalents) is important for alternative energy quenching as well as the mechanism between photorespiration and rapidly reversible non-photochemical quenching. These relationships should be examined both in the laboratory and under more field-like conditions in model and crop species.

### Photorespiration as a marker and key trait for whole-plant stress resilience

It is clear that an increased photorespiratory rate under drought and high temperature conditions is a consequence of enzyme kinetics and plant physiological responses, yet unclear if there is increased stress resilience due to changes in photorespiration. Kinetically, rubisco specificity for carbon dioxide relative to oxygen (carboxylation:oxygenation 4:1 at 5° C (Viil et al. 2012)) decreases with temperature (carboxylation:oxygenation 1:1 at 41° C (Viil et al. 2012)), increasing photorespiratory pressure under hotter conditions (Badger and Collatz 1978). Physiologically, under heat and drought stress, stomata close. Consequently, carbon dioxide is consumed within the intercellular airspace and replaced more slowly due to higher diffusional resistance. While the net effect of this on net CO_2_ assimilation is actually moderated by stomatal conductance (discussed in more detail in Busch (2020)), relative rates of photorespiration increase substantially, highlighting a potential link specifically to plant resilience. In addition, the CO_2_ released from photorespiration can help maintain rubisco catalysis (Igamberdiev 2015). Could these kinetic and physiological responses be a feature, not a bug, of heat and drought stress response?

This question has been approached through measuring two abundant, easily detectable, and key photorespiratory intermediates: serine and glycine. In a major effort to determine which metabolites correlate with grain yield in *Zea mays* under drought and heat stress, serine and glycine were often significantly negatively correlated with yield (Obata et al. 2015). Glycine concentration was also different in a drought-resistant barley line, and this mapped to a quantitative trait locus, indicating there may be direct or indirect genetic control (Templer et al. 2017). In at least one study, serine correlated with yield loss in drought-stressed (but not unstressed) rice (Melandri et al. 2019). These studies must be interpreted with the caveat that flux does not necessarily correlate with pool size, only that there are potential links between a metabolite involved in photorespiration and stress resilience.

Interestingly, photorespiration can have metabolic effects on non-photosynthetic tissues through metabolite translocation. For example, when photorespiration was targeted for genetic improvement, there were large changes in the root metabolome (Timm et al. 2025). These findings raise the question of how photorespiration can impact not only photosynthetic tissues, but also other structures important for yield such as tubers following translocation of photorespiratory-linked metabolites.

These studies also raise the question of whether it is necessary for photorespiratory capacity to adapt or acclimate to meet increased demand imposed by warmer climates. In this area, the evidence is mixed. In support of altered capacity to facilitate higher flux are (i) that protein abundance of photorespiratory proteins increased in response to low water and low nitrogen in field-grown wheat (Kang et al. 2023) and (ii) the activity of some photorespiratory enzymes (phosphoglycolate phosphatase and catalase) was higher in the heat-adapted *Rhazya stricta* as compared to the warm-adapted *Nicotiana tobacum* (Gregory et al. 2023). Other work shows no relationship; for example, there was no change in measured enzyme activities in paper birch exposed to combinations of high temperature and CO_2_ (Gregory et al. 2024). These conflicting results suggest that the need to acclimate photorespiration may be context and species dependent, but this could mean that there is a more complicated relationship between plant resilience and photorespiration. Parsing the alternative fluxes from photorespiration to central metabolism would improve understanding the nature of this relationship.

### Photorespiration in the metabolic context

**Fig. 2:**
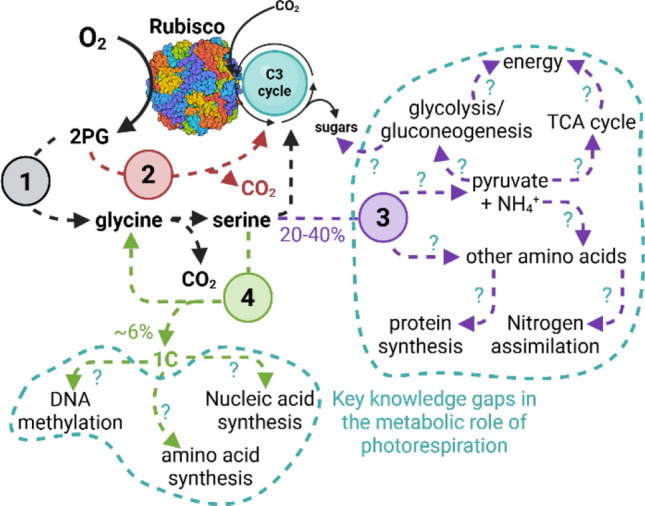
The many routes of carbon following rubisco oxygenation through metabolism. Shown are the “canonical” route through the chloroplast, peroxisome, and mitochondria (1), additional pathways that have similar end products as discussed in the text (2), removal of serine to downstream metabolism other than photorespiration (3), and the non-photorespiratory production of 1-carbon units (1C) during serine to glycine conversion (4). Also shown are potential downstream fates of 1 C units and serine through metabolism. Given current limitations of INST-MFA to penetrate secondary and specialized metabolism, Genome-Scale Metabolic Models (GSMMs) are ideal tools for integrating isotope tracing datasets and evaluating metabolite fate

Photorespiration is often depicted as a single cycle isolated from the rest of metabolism, where 2PG enters and 3PG is regenerated into the C3 cycle with the release of carbon dioxide and cycling of amino groups (Fig. 2). There is growing evidence, however, that a large fraction of these photorespiratory intermediates do not pass through this canonical pathway. Sometimes these alternative fluxes involve similar reactions and intermediates as canonical photorespiration, such as the cytosolic overflow of hydroxypyruvate reduction (Timm et al. 2008; Timm et al. 2011), proposed cytosolic glyoxylate shunt (Jiang et al. 2025), chloroplastic glycolate processing (Bismarck et al. 2023), or the proposed complete decarboxylation of glyoxylate in conifers (Miyazawa et al. 2023). These alternative pathways differ from canonical photorespiration but result in the same final products of 3PG and carbon dioxide.

Recent evidence suggests that not all carbon in 2PG is recycled as 3PG or released as carbon dioxide. For example, it has been hypothesized from the oxygen-insensitivity of the response of carbon assimilation to carbon dioxide concentrations, that large amounts of serine and possibly some glycine leave photorespiration (Sharkey 1985; Harley and Sharkey 1991). Until recently, supporting evidence was limited to high-light and conditions approaching carbon saturation. Recent ^13^CO_2_ labeling work supports that 20–40% of photorespiratory carbon leaves the photorespiration pathway as serine and not glycine (Fu et al. 2023). This is consistent with other work using ^13^C and ^15^N metabolite tracers supporting that glycine is maintained in photorespiration, but is inconsistent with modeling work from this same study suggesting that serine export from photorespiration could be very low (Abadie et al. 2016). Regardless of serine export, ^13^C labeling demonstrates that photorespiration is a large contributor to one-carbon (1C) metabolism, with a 90% decrease in 1 C flux when photorespiration was minimized by decreasing oxygen concentration in *Nicotiana tabacum* leaves (Gashu et al. 2025). The link between photorespiration and 1 C metabolism appears to come from cytosolic interconversion of serine to glycine via serine hydroxymethyl transferase. Contrasting with these results, there appears to be substantial non-photorespiratory 1 C production at least in poplar leaves (Jardine et al. 2024). There are multiple differences in the methodologies employed by these studies including the species examined, methods used to measure the flux, how photorespiration was minimized, and the time of exposure to the conditions designed to limit photorespiration. Future work could evaluate more broadly when 1C metabolism relies on photorespiration across multiple species and how well C1 metabolism can adapt to changing climate conditions driving different rates of photorespiratory flux.

These carbon outlets from photorespiration could explain a variety of relationships between photorespiration and plant nutrition. For example, when glycine and serine leave photorespiration, their amino groups are removed from the pool used for the aminotransferase reactions of the peroxisome that perpetuate canonical photorespiration. The removal of these amino groups necessitates additional groups to maintain photorespiratory flux (Busch et al. 2018). This could explain the positive correlation between photorespiration and nitrate assimilation (Bloom et al. 2002; Bloom et al. 2010; Bloom et al. 2014; Ab and Aj 2003; Cousins and Bloom 2004; Rachmilevitch et al. 2004). These links could also help explain why crops grown under elevated carbon dioxide have reduced protein content and vitamers involved in one-carbon metabolism (Myers et al. 2014; Zhu, et al. 2018).

So, does this relationship between photorespiration and plant nutrition explain all the fate of serine and glycine that leaves photorespiration? To estimate what possible metabolic sinks could explain such large rates of serine removal, calculations of the total needed supply of photorespiratory serine can be compared to the various sinks of serine in metabolism. We will focus on serine since there is the most evidence for serine removal from gas exchange and ^13^C labeling studies. These calculations can also be used to determine what portion of nitrate assimilation is needed to support rates of serine removal from photorespiration.

Estimates of serine production from photorespiration can be made starting with whole-plant CO_2_ gas exchange assimilation (*A*) of Arabidopsis rosettes, which averaged 7 μmol CO^2^ m^−2^ s^−1^ (Kölling et al. 2015). From this value of *A*, rates of rubisco oxygenation (*v*_*o*_) can be estimated using the fundamental representation (Eq. 1) of the Farquhar, Berry, von Caemmerer (FvCB) (Farquhar et al. 1980; Caemmerer and Farquhar 1981) model of photosynthetic gas exchange where

1 $$A= {v}_{c}-0.5{v}_{o}-{R}_{L}$$

and *v*_*c*_*, and R*_*L*_*,* represent rates of rubisco carboxylation and respiration in the light, respectively. Assuming a commonly measured *v*_*o*_*:v*_*c*_ of 0.2 and *R*_*L*_ of 0.35 μmol CO^2^ m^−2^ s^−1 3^, (Ma et al. 2014; Xu et al. 2021; Walker et al. 2013), 12 h of illumination, and 32 mg fresh weight cm^−2^, this results in total daily carbon fixation of 945 μmol Carbon fixed by rubisco g^−1^ FW day^−1^ and 220 μmol oxygen fixed by rubisco g^−1^ FW day^−1^. A 20–40% proportion of photosynthetic glycolate carbon to serine would therefore translate to 29–59 μmol serine g^−1^ FW day^−1^ since each oxygenation reaction moves two carbons into photorespiration as glycolate and a serine has three carbons total. This rate of serine removal, on a nitrogen basis, from photorespiration is on the same order as measured rates of de novo nitrate assimilation, which is between 19 and 85 μmol g^−1^ FW day^−1 46^. The implications of this are that a large portion of nitrate assimilation could go to replace amino groups lost from photorespiration due to removal of serine.

The fate of serine removed from photorespiration is unknown, and it is not clear if rates of de novo protein synthesis can account for this serine. To determine if rates of protein synthesis are large enough to serve as the “sink” of serine removal from photorespiration, we will approximate rates of protein synthesis on a fresh weight basis. Protein accumulation in Arabidopsis leaves averages 0.03–0.15-fold change daily, depending on growth rate (Li et al. 2017). We assumed a 0.1-fold daily increase as an average and 20 mg g^−1^ FW total protein (based on ~ 2% protein on a FW basis), which translates to 2 mg g^−1^ FW d^−1^ of protein synthesis. Since Arabidopsis leaf proteins are 5.5% serine by weight, this results in 1.05 μmol serine g^−1^ FW day^−1^. This rate only accounts for 3% of the lowest rate of serine export estimated above, but there are several other protein-specific factors to consider. Notably, rates of protein synthesis in the light are threefold larger than those in the day (Duncan and Millar 2022) and serine is also used for cysteine (1.9% of protein content) and methionine (2.4%), so the total demand for serine and serine-derived amino acids in the light could be as high as 5.0 μmol serine g^−1^ FW day^−1^ under illumination, which could account for 9–17% of all serine exported from photorespiration assuming it totals to the 29–59 μmol serine g^−1^ FW day^−1^ estimated above. Note that the calculations above account for export on a carbon and not nitrogen basis. While in some situations these will be tightly linked, they will not be so if amino groups are recovered from serine after carbon export, as with what happens during the aminotransferase reactions of photorespiration.

These calculations indicate that protein synthesis alone can’t account for all serine predicted to leave photorespiration. This could be due to inaccurate estimates of serine removal or additional fates for serine removed from photorespiration. These fates could include lipid headgroup synthesis through ethanolamine (Timm et al. 2021), activities of promiscuous aminotransferases inside or outside of the peroxisome (Koper et al. 2022), or as yet to be described pathways. Resolving the fate of this serine is challenging, due in part to the limitations in the approaches used to estimate its removal.

The large rates of serine removal mentioned above come from fitting the kinetics of ^13^C labeling during ^13^CO_2_ feeding to resolve metabolic fluxes in an approach known as isotopically nonstationary metabolic flux analysis (INST-MFA) (Ma et al. 2014; Cheah and Young 2018). While a powerful approach for resolving fluxes through central metabolism, INST-MFA is limited in resolving metabolic fluxes that are sufficiently labeled during the ^13^CO_2_ feeding. Since many specialized and nutrition-related compounds are very slowly labeled during the < 1 h feeding experiment, the fate of photorespiratory carbon remains unresolved, suggesting that alternative methods are needed to understand the fate of serine removed from photorespiration.

### Genome-scale resource models can bring clarity to the relationship between photorespiration, plant nutrition, and resilience

Limited penetration of ^13^C into secondary metabolism through relatively short-term INST-MFA studies necessitates other tools to examine the fate of photorespiratory carbon. Genome-scale models of metabolism (abbreviated GSMMs, GSMs, or GEMs) will be key tools in this investigation, since they have been recently used to track metabolite origin and fate a model developed with isotope tracking data (^13^C MFA) that did not extend to the tracked metabolite (pyrophosphate) (Schroeder et al. 2023). GSMMs are matrix-based, linear, and strictly stoichiometric representations of the biochemical repertoire of an organism (Orth et al. 2010; Thiele and Palsson 2010). This repertoire is derived primarily from genomic evidence of biochemical functions (annotations, bioinformatics analysis, synteny, etc.), with additional reactions as evidenced by experiments, demanded by necessity of the metabolic network, and simulating organism growth and maintenance (Orth et al. 2010; Thiele and Palsson 2010; Schroeder and Saha 2020a). The GSMM network is then interrogated through optimization-based techniques since this network has many degrees of freedom. The most common analysis tool is Flux Balance Analysis (FBA), which predicts flux distribution through the metabolic network using constraints based on organism growth, nutrient availability, and/or measured nutrient uptake rates and an assumed cellular objective (Orth et al. 2010). Predictions made from FBA tend to be overly optimistic, accounting only for stoichiometry and therefore missing other limiting factors within metabolism such as kinetics, regulation, and protein expression levels, which would generally result in lower reaction rates. These can be extended to resource allocation models (RAMs) which constrain reaction rates by enzyme turnover number and abundance, overcoming one of these limitations (Schroeder et al. 2024). These limitations can be further eroded through kinetic models of metabolism. However, kinetic models have three key limitations. First, reaction kinetics are often unmeasured in vivo, necessitating computationally difficult (or intractable) and data-intensive tools to estimate kinetic parameters (Hu et al. 2024; Choudhury et al. 2024; Gopalakrishnan et al. 2020; Foster et al. 2021). Second, these models use relative concentration, and therefore kinetic parameters are also relative (Hu et al. 2024; Choudhury et al. 2024; Gopalakrishnan et al. 2020; Foster et al. 2021). Third, kinetic models are non-linear, becoming computationally prohibitive to run beyond about 500 reactions (Hu et al. 2024; Choudhury et al. 2024; Gopalakrishnan et al. 2020; Foster et al. 2021). Therefore, genome-scale kinetic models of plant species have not yet been publicly released.

GSMMs and RAMs have been used to model a number of plant species including Arabidopsis (Saha et al. 2011; Wendering et al. 2025; Schroeder and Saha 2020b), maize (Saha et al. 2011; Chowdhury, et al. 2023), tomato (Yuan et al. 2016), black cottonwood (Barbosa, et al. 2024), and rice (Chowdhury et al. 2025), investigating metabolic effects of temperature, drought, and lifecycle stage, among other phenomena. Despite these applications, GSMMs often fail to consider photorespiration (Schroeder and Saha 2020b; Ravindran et al. 2024) (even when temperature stress is a key concern (Chowdhury, et al. 2023)) or appear to have only a partial understanding of the phenomena, describing photorespiration as a “condition” (Yuan et al. 2016), “state” (Saha et al. 2011), or fixed ratio regardless of growth conditions (Barbosa, et al. 2024). One notable exception is the recent (2025) ecAraCore model (Wendering et al. 2025), which derives three constraints from the FvCB model (Caemmerer et al. 2025). However, overall, current GSMMs are not well equipped to investigate the role of photorespiration in wider metabolism despite their clear potential.

Curating existing GSMMs to ensure accurate photorespiratory pathways and incorporating biochemical models like FvCB will be key to tracking photorespiratory carbon through plant metabolism and re-envisioning the role of photorespiration as an integral part of C3 plant metabolism. These models can be interrogated for fitness differences between “closed loop” (e.g. all 2PG carbon to 3PG or CO_2_) and “open loop” (serine and glycine can leave) photorespiration to investigate if or how photorespiration can provide a metabolic advantage under stress conditions beyond cofactor recycling. GSMMs can further be used to interrogate ATP to NADH consumption ratios under various growth and stress conditions. Similarly, GSMMs can be used to investigate maximal rates of ATP and reducing equivalent dissipation across all metabolism by photorespiration to test its feasibility as protection against excess energy or electrons. Expanding on GSMMs, certain types of RAMs may be key to elucidating serine and glycine fate, as they can give a comprehensive accounting of amino acid fate beyond a biomass equation through modeling enzyme activity, synthesis, and partitioning. Given the central role of rubisco kinetics in the partitioning of its activity, a modified RAM modeling framework which incorporates select enzyme and transport kinetics (particularly photorespiration, photosynthesis, and known bottlenecks like the chloroplastic phosphate pool) informed by the FvCB model and subsequent work (Sharkey 1985; Harley and Sharkey 1991) would be key to a new generation of genome-scale plant models capable of interrogating key questions surrounding photorespiration.

### Future directions for determining metabolic fluxes

There are many key questions that the above discussion presents to better integrate photorespiration with other aspects of plant function. One fruitful area for future work will be using high-confidence flux solutions for central metabolism to constrain genome-scale resource allocation models (RAMs) to determine the fate of intermediates that leave photorespiration into specialized metabolism. This work would be especially important in crop plants under near-field conditions; only one of ten INST-MFA (or INST-MFA-like) studies has examined a staple food crop or conditions outside of a growth chamber and none have been done under drought or high temperatures. This point is illustrated by the compilation of sampling and experimental conditions outlined in Table 1. Another poorly resolved area is how photorespiration affects whole-plant physiology and seed nutrition; in other words, do leaf-level changes in metabolic flux at the leaf level alter seed composition? These fluxes are especially important to understand in the context of re-designing photorespiratory metabolism, especially given the proliferation of potential synthetic “bypass” designs. Resolving how photorespiratory fluxes influence whole-plant physiology and seed composition—especially under realistic environmental conditions—will be essential for integrating photorespiration into a predictive and engineering framework of plant resilience and nutritional quality.

**Table 1:** Mass flux analysis studies on leaf metabolism. Information on the species, growth, and sampling conditions included below

*Species*	*Growth conditions*	*Sampling conditions*	*Citation*
*Arabidopsis thaliana*	Growth Chamber low light (120 µmol m^−2^ s^−1^)	Similar to growth	(Szecowka et al. 2013)
*Arabidopsis thaliana*	Growth Chamber low light (200 µmol m^−2^ s^−1^)	Low (200 µmol⋅m^−2^⋅s^−1^) and higher (500 µmol⋅m^−2^⋅s^−1^) light	(Ma et al. 2014)
*Arabidopsis thaliana*	Growth Chamber low light (100 µmol m^−2^ s^−1^)	Similar to growth but at 2% and 21% O_2_	(Gashu et al. 2025)
*Camelina sativa*	Growth Chamber higher (500 µmol m^−2^ s^−1^) light	Similar to growth	(Xu et al. 2021)
*Camelina sativa*	Growth Chamber higher (500 µmol m^−2^ s^−1^) light, long and short days	Similar to growth	(Xu et al. 2023)
*Camelina sativa*	Growth Chamber higher (500 µmol m^−2^ s^−1^) light	Similar to growth and high light/high CO_2_	(Xu et al. 2025)
*Camelina sativa*	Growth Chamber higher (500 µmol m^−2^ s^−1^) light, long and short days	Similar to growth	(Xu et al. 2022)
*Zea mays*	Growth Chamber higher (500 µmol m^−2^ s^−1^) light	Low (160) and high (500 µmol m^−2^ s^−1^) light	(Medeiros et al. 2022)
*Nicotiana tabacum*	Greenhouse (> 500 µmol m^−2^ s^−1^)	Similar to growth	(Chu et al. 2022)
*Nicotiana tabacum*	Growth Chamber low light (100 µmol m^−2^ s^−1^)	Similar to growth but at 2, 21, and 40% O_2_	(Fu et al. 2023)

## Data Availability

There is no original data presented as part of this manuscript.
